# Bedtime Oral Hygiene Behaviours, Dietary Habits and Children’s Dental Health

**DOI:** 10.3390/children8050416

**Published:** 2021-05-19

**Authors:** George Kitsaras, Michaela Goodwin, Michael P. Kelly, Iain A. Pretty

**Affiliations:** 1 Dental Health Unit, Division of Dentistry, The University of Manchester, Manchester M1 5GH, UK; michaela.goodwin@manchester.ac.uk (M.G.); iain.a.pretty@manchester.ac.uk (I.A.P.); 2 Department of Public Health and Primary Care, University of Cambridge, Cambridge CB2 0SR, UK; mk744@medschl.cam.ac.uk

**Keywords:** early childhood caries, free sugars, diet, psychosocial, child, wellbeing, prevention, decay

## Abstract

*Background:* Oral hygiene behaviours as well as dietary habits before bed can affect children’s dental health resulting in higher prevalence of dental disease. Dental disease can affect children’s health, development and even school performance. If left untreated, dental disease can progress and it can lead to extractions under general anaesthetic causing further distress for children and families. Consistent and appropriate oral hygiene behaviours and dietary habits can prevent dental diseases from occurring in the first place. *Objective:* This cross-sectional study examines the relationship between oral hygiene behaviours, dietary habits around bedtime and children’s dental health. *Methods:* A total of 185 parents with children between the ages of 3 and 7 years from deprived areas participated in the study. Data on bedtime routine activities were collected using an automated text-survey system. Children’s dental health status was established through examination of dental charts and dmft (decayed, missed, filled teeth) scores. *Results*: In total, 52.4% of parents reported that their children’s teeth were brushed every night. The majority of children (58.9%) had dmft scores over zero. In total, 51 (46.7% of children with dmft score over 0 and 27.5% of all children) children had active decay. The mean dmft score for those experiencing decay was 2.96 (SD = 2.22) with an overall mean dmft score of 1.75 (SD = 2.24). There were significant correlations between frequency of tooth brushing, frequency of snacks/drinks before bed and dmft scores (r = −0.584, *p* < 0.001 and r = 0.547, *p* = 0.001 respectively). Finally, higher brushing frequency was associated with a lower likelihood of a dmft score greater than 0 (Exp(B) = 0.9). *Conclusions:* Despite families implementing oral hygiene behaviours as part of their bedtime routines those behaviours varied in their consistency. Results of this study highlight the need for additional studies that consider bedtime routine-related activities and especially the combined effects of oral hygiene practices and dietary habits due to their potentially important relationship with children’s dental health.

## 1. Introduction

Dental caries is the most common preventable disease with an estimated 60% of children worldwide experiencing dental caries that can often lead to pain, discomfort and may impact negatively on quality of life [1]. Dental caries is a multifactorial disease that starts on a microbiological level and is affected by salivary flow and composition, exposure to fluoride, consumption of dietary sugars and by preventive behaviours (e.g., brushing teeth) [2]. In early stages, dental caries can be reversed but without proper care, can progress until the tooth requires extraction [2]. Oral diseases during childhood can have a negative impact on the life of preschool children and their parents [3]. The negative impact of dental caries at an early age includes chewing difficulties, sleeping difficulties, changes in behaviour (e.g., irritability), implications with psychological development (such as low self-esteem), loss of school days with subsequent decrease in school performance [3,4,5]. Moreover, dental caries, if left untreated, can lead to dental extractions under general anaesthetic with further implications for children’s psychosocial wellbeing with increased pain, need for hospitalisation and increased anxiety for children and parents [6]. Also, dental extractions under general anaesthetic add pressures to public finances and healthcare services [6].

Bedtime is a particularly important time for both diet and preventive behaviours with regards to dental health [7,8]. Consumption of food and drink, especially those, containing free sugars at bedtime is an important risk factor for caries due to decreased salivary flow shifting the balance toward demineralisation rather than remineralisation [9]. Available guidelines, public organisations and bodies (including the National Health Service in the UK) emphasise the importance of parental supervised brushing (PSB) twice daily with fluoridated toothpaste, with one of the occasions being before bedtime, as well as controlling dietary sugars’ intake especially at bedtime [8,9,10]. Both control of free sugar intake, especially before bed, and twice daily brushing rely on the development and maintenance of healthy family routines [11]. Apart from dental health, bedtime routines, as a recurrent family behaviour, have shown important associations with key child wellbeing and developmental areas including quality of sleep, school readiness, school performance, psychosocial development as well as family functioning and parental wellbeing [12,13,14].

Currently, there is little information on the characteristics of bedtime routines in families with young children and in particular, with regard to oral hygiene and dietary behaviours before bed. Given the importance of bedtime routine behaviours on children’s dental health, it is crucial to understand what occurs within households and how different behaviours can affect children’s dental health. Once this issue is explored, we can then consider possible ways of supporting families to achieve better routines and by extension, better dental health for their children.

## 2. Objective

The main objective of this study is to gain a better insight into bedtime oral hygiene behaviours and dietary habits in families with young children and the effect these behaviours might have on prevalence of dental disease in children.

## 3. Methods

### 3.1. Recruitment

A total of 200 parents with young children were recruited for this study. Recruitment took place between February and July 2018 in the United Kingdom (UK). Participants were approached during routine dental appointments in four areas around the UK. Initial approach was made by staff at the dental practices. A member of the research team provided additional information to parents if they were interested in the study. Exclusion criteria included: (a) inability to read and speak English, (b) not having access to a working mobile phone and (c) not having children between the ages of 3 and 7. Informed consent was also obtained. Participants were compensated for their participation in the study with a GBP 10 shopping voucher. All aspects of the study were approved by the Health Research Authority (HRA) in England (Integrated Research Application System (IRAS) ID: 238552).

### 3.2. Data Collection

Data were collected on (a) family bedtime routine activities, (b) children’s dental health and (c) sociodemographic information. In terms of assessing bedtime routine activities, data collection took place over a 7-night period. This approach allowed for a wider range of data including weekday and weekend activities that took place in each household. In line with a previous study [14], an automated text-survey system delivered directly to each participant’s mobile phone was utilised for assessing bedtime routines. The automated text-survey included both open ended and closed questions. There were specific questions on oral hygiene behaviours (i.e., if they brushed their teeth that night and who brushed the children’s teeth, etc.) and questions on dietary habits the hour before bed. Children’s dental health status was assessed through dental charts completed during routine dental appointments at general dental practices by dental professionals. During informed consent, participants granted permission to request access to copies of their children’s dental charts. Copies of dental charts were requested at the end of the study. The charts reflected the dental health status of the children as seen on their routine appointment when recruitment took place. Sociodemographic information was collected through a brief demographic questionnaire at the point of recruitment. Index of Multiple Deprivation (IMD) scores were calculated based on participants’ addresses. IMD is a commonly used metric of deprivation in England with higher scores indicating higher deprivation.

### 3.3. Data Analysis

At the end of data collection, all data relating to bedtime routines were coded and analysed using SPSS (IBM SPSS Statistics for Macintosh, Version 25.0). For dental health status, dmft (decay, missed, filled teeth (primary teeth)) scores were utilised. Scores over 0 indicated presence of decayed, missed and/or filled teeth. Data were descriptively analysed in order to examine frequency and prevalence of bedtime routine activities. Since not all families responded to every night of the assessment, percentage scores were used instead of raw scores to reflect frequencies. A correlation analysis was used to examine the relationship between tooth brushing, dietary habits before bed and dmft scores. Finally, a binary regression analysis was performed to explore which of the key variables relates to having a dmft score over zero.

## 4. Results

### 4.1. Sample Characteristics

From the 200 parents recruited a total of 185 (92% retention rate) completed data collection. Parents had a mean age of 34.6 (SD = 5.01) with the majority being females with only 24 male parents participating in the study. Most families had only 1 child (65%) while only 5% of the families had more than 3 children. In terms of children’s characteristics, just over half (56.8%) were males. In total, 89 parents (48.1% of the sample) were of white ethnic background, 73 (39.5%) were of Asian/British-Asian background and 23 (12.4%) were of Black/Black-British/Caribbean ethnic background. The vast majority of participants (77.8%) lived in deprived areas with an overall mean IMD score of 41.83 (SD = 16.43) and a maximum IMD score of 79.65. Most parents (*n* = 118, 63.8%) were high school graduates, a total of 54 parents (29.2%) have post-high school qualifications with 13 parents (7%) reporting university degrees. Most parents were stay at home parents (N = 75, 40.5%), in total, 55 parents (29.7%) worked part-time, 36 (19.5%) worked full-time, 13 parents classed themselves as students of continuing education (7%) and finally, 6 parents were unemployed at the time of the study (3.2%).

### 4.2. Bedtime Oral Hygiene Behaviours and Diet

Most parents (N = 183, 98.9% of parents) reported brushing their children’s teeth at least one night a week with 97 parents (52.5%) reported brushing their children’s teeth every night of the week. A total of 2 parents (1.1%) reported not brushing their children’s teeth at all at night. Table 1 summarises the frequency of brushing teeth before bed. In terms of who brushed the children’s teeth, there was an almost equal distribution among parents brushing their children’s teeth (34.1%), parents supervising their children while children brush their teeth (31.9%) and children brushing their teeth with no supervision or assistance (34%). When brushing children’s teeth, most parents (N = 173, 94.5%) reported using fluoride toothpaste. Most parents (N = 153, 83.6%) reported brushing for at least 2 min each night.

Only 8 (4.3%) of participants reported completely avoiding snacks and/or drinks the hour before bed, 82 (44.3%) of participants allowed only water and/or unflavoured milk the hour before bed while 95 (51.3%) of participants reported allowing snacks/drinks other than water and/or unflavoured milk the hour before bed. From the 95 parents who allowed food/drinks before bed, 36 (37.9%) allowed them every night of the week with the remaining families (N = 59, 62.1%) allowing snacks/drinks at least 4 nights a week. There was an overall increase in snacks/drinks on Friday and Saturday nights with most families reporting allowing such foods before bedtime (N = 80, 84/2%). From those who allowed food and/or drinks in the hour before bed, 42 (44.3%) parents allowed consumption of fruit or vegetables excluding fruit juices, purees and other snacks/drinks where fruits/vegetables have been processed while 53 (55.7%) of parents allowed sugary or savoury snacks including chocolate, crisps, fruit juices and/or soft drinks.

### 4.3. Dental Health Status

The majority of children (N = 109, 58.9%) had dmft scores over zero with a maximum dmft score of 13 (M = 2.96, SD = 2.22). The mean decay score was 1.10 (SD = 1.57) while the mean filled score was 1.86 (SD = 1.65). In total, 51 (46.7% of children with dmft score over 0 and 27.5% of all children) children had active decay. The mean dmft score for those experiencing decay was 2.96 (SD = 2.22) with an overall mean dmft score of 1.75 (SD = 2.24). The majority of dmft scores ranged from 1 to 4 in both decay (49.5% of cases) and filled (77% of cases) components. Due to issues with the information provided from the dental charts, no information was available regarding missed teeth. Bivariate correlations showed no significant associations on IMD scores or children’s age with regards to dmft scores. A Kruskal–Wallis analysis showed no significant differences with regards to ethnicity and dmft scores.

### 4.4. Relationship between Bedtimes and Dental Health

A bivariate Pearson correlation revealed a significant negative correlation between dmft scores and percentage of nights brushing teeth r = −0.584, *p* < 0.001 showing that less frequent tooth brushing at night is associated with higher dmft scores. Additionally, there is a significant positive correlation between dmft scores and percentage of nights having snack and/or drink before bed r = 0.547, *p* = 0.001.

Binary logistic regressions were performed to examine the relationship among key variables and dmft scores. IMD scores, children’s age, frequency of brushing teeth before bed and frequency of having snack and/or drinks before bed were included in the model. The logistic regression model was statistically significant χ^2^(4) = 60.994, *p* < 0.001. The model explained 37.9% (Nagelkerke R^2^) of the variance in dmft scores over 0 and correctly classified 75.7% of the cases. As brushing frequency increased, the likelihood of having a dmft score over 0 decreased by 0.9. Increasing age was also associated with higher likelihood in having a dmft score higher than 0. There was no significant effect with regard to frequency of nights having food/drinks the hour before bed (excl. water and unflavoured milk and fruit/vegetables) and IMD scores. No assumptions were violated. A second binary logistical regression was performed on the frequency of snacks/drinks before bed and dmft scores. The results were nonsignificant (*p* = 0.068). Table 2 summarises the results of the binary logistical regressions in predicting dmft scores over 0.

## 5. Discussion

This study examined oral hygiene behaviours and dietary habits before bed in families with young children and their relationship with children’s dental health. As with previous research, the results of this study showed the importance of bedtime oral hygiene and dietary habits in relation to children’s dental health. The results from the study showed that despite families implementing oral hygiene behaviours during bedtime those varied and could have been improved in accordance with available guidelines and recommendations. While a small majority (52.4%) of participants reported that their children’s teeth were brushed every night, there was a significant number of parents reporting not brushing every night of the week with a marginal 1.1% not brushing teeth at all before bed. In terms of dental health status, the majority of children participating in this study (27.5%) presented with obvious decay experience as calculated by their dmft scores. This score is in line with the national average (23.3%) for England based on the National Dental Epidemiology Programme [15]

Results from the analysis showed that there is a mixed picture with regard to who brushed the children’s teeth with around a third of children brushing their teeth alone without any supervision. That mixed picture is reflected in national average data from the United Kingdom regarding unsupervised toothbrushing [16]. Unsupervised tooth brushing can have detrimental effects on dental health status in young children and despite available recommendations a large proportion of children (50% of 5-year-olds) brush their teeth without any adult supervision [16]. Different factors have been identified as important in terms of not following good practice recommendations and not translating evidence-based guidelines such as supervised tooth brushing into daily practice. Despite most parents being aware of the importance of tooth brushing, issues relating to children’s resistance, lack of resources as well as lack of behavioural management techniques from the parent’s perspective result in problematic implementation of good practices on oral care [17,18]. Moreover, around a fifth of those who reported brushing their children’s teeth did so for less than the recommended 2 min. Brushing teeth with a fluoride toothpaste for 2 min two times a day is recommended good practice by various bodies and organisations including the National Health Service (NHS) in the UK. Duration, frequency and brushing techniques are all vital aspects of preventive oral hygiene care especially for young children who are in the process of acquiring these critical skills for later life [19]. Learning these skills right at an early age, can help children maintain good oral hygiene practices in later life leading to lower risk of developing dental disease.

Apart from tooth brushing, dietary habits before bed, especially consumption of food and/or drinks with their associated free sugars, is another important factor for children’s dental health [20]. In this study, there was a significant association correlation between dmft scores and percentage of nights having snack and/or drink before bed. Nevertheless, percentage of nights having a snack/drink before bed (excl. water, unflavoured milk and fruit/vegetable) did not significantly increase the risk of a dmft score over 0. Dietary links to dental health have been long-established from a physiological and biological perspective with good evidence regarding the importance of avoiding free sugars in general [21]. Dietary habits the hour before bed could be a potential proxy measure of overall dietary and snacking habits in young children [10]. Results from this study showed that the majority of parents allowed their children to have snacks and/or drinks before bed. Water, unflavoured milk as well as fruit and vegetables where the cellular structure has not broken down (i.e., puree, blended etc.) are treated as natural sugars with limited implications on dental health [8]. On the other hand, free sugars (i.e., natural sugars which have been broken down as well as added sugars in food and drinks) have shown damaging effects on dental health due to their cariogenic load [8]. Limiting exposure to free sugars throughout the day and especially before bed is vital in ensuring good oral health for children, weight control and appropriate, nutritious diet. At the moment, a range of public bodies and initiatives including the Change4Life campaign in England from Public Health England and the NHS focus on promoting better snacking options for children, limit amount of sugar intake and promote overall nutrition [22].

Establishment of good oral hygiene behaviours and dietary habits early in life is important in achieving long-term good dental and avoiding dental caries and decay from an early age [2]. Avoidance of early childhood caries is crucial given the important link between early childhood caries and later dental health status in adolescents and adults [2]. Bedtime presents a unique opportunity given the importance of bedtime brushing and bedtime dietary habits for children’s dental health. Consistent routines that encompass beneficial, repetitive, health behaviours such as brushing teeth are vital in ensuring adequate protection against dental disease [23]. Having a good bedtime routine in place is not panacea to all problems but investing in support for parents to achieve better bedtime routines can allow for essential behaviours for children’s health and wellbeing to occur. Additionally, the repetitive nature of bedtime routines and the emphasis on routines from an early age can help children adopt beneficial behaviours quickly and sustain those behaviours over time. These beneficial behaviours, including behaviours around oral health, can trickle down to other times of the day for example, in morning routines that are also very important for achieving good oral hygiene practices and avoiding dental disease. Based on the results of this study, it is clear that most parents know what they need to do with some of them achieving a good routine most nights of the week. However, there are clear discrepancies in the frequency and robustness of oral hygiene and dietary behaviours that need to be addressed.

## 6. Limitations

One key limitation can be found in the use of dmft scores through the examination of routinely available dental data from general dental practices. This approach, despite being cost-effective and timely, cannot provide an in-depth examination of the dental health status of the children potentially limiting our overall understanding. The dental charts assessment was not able to identify if missing teeth were a result of extractions or lost naturally. Additionally, due to the fairly small sample, the nature of the dental data provided and the cross-sectional design it is not possible to assume causality with regard to bedtime oral health hygiene behaviours, dietary habits and children’s dental health. Finally, the bedtime routine questionnaire and the repeated nature of its collection, could have led to an unintended change in families’ routines during the study resulting in distorted data with regard to the quality of bedtime routines. However, any desirability bias effect caused by repeated measurements might have been averted due to the quick and non-intrusive nature of the questionnaire that was delivered through text messages.

## 7. Conclusions

This study highlighted the existing discrepancies in the characteristics of bedtime routine activities relating to oral hygiene behaviours and dietary habits before bed. It also highlighted the possible effect these discrepancies can have on children’s dental health. Bedtime-related activities, including oral hygiene behaviours, present an important area for further exploration given their relationship with key child health, wellbeing and development areas. Promotion, establishment and maintenance of optimal bedtimes including good oral hygiene behaviours and dietary habits before bed is a crucial first step for successful short- and long-term oral health outcomes.

## Figures and Tables

**Table 1:** Frequency of brushing teeth before bed.

Activity	Achieved Every Night		Never Performed		Achieved at Least 50% of Nights		Achieved Less than 50% of Nights	
	N	%	N	%	N	%	N	%
Brushing before bed	97	52.5	2	1.1	61	33	25	13.5

**Table 2:** Binary logistical regression results.

Predictor	B	S.E.	Sig.	Exp (B)
Age of child	0.237	0.120	0.048	1.267
Percentage of nights brushing teeth	−0.100	0.018	0.000	0.905
Percentage of nights having snacks/drinks	0.009	0.006	0.155	1.009
IMD score	0.001	0.011	0.937	1.001

B: values for the logistic regression equation for predicting the dependent variable from the independent variable; S.E.: standard errors associated with the coefficients; Sig.: level of significance; Exp (B): odd ratios for the predictors.

## Data Availability

The data presented in this study are available in within the article. The data presented in this study are available on request from the corresponding author.
